# Expression of Calretinin Among Different Neurochemical Classes of Interneuron in the Superficial Dorsal Horn of the Mouse Spinal Cord

**DOI:** 10.1016/j.neuroscience.2018.12.009

**Published:** 2019-02-01

**Authors:** Maria Gutierrez-Mecinas, Olivia Davis, Erika Polgár, Mahvish Shahzad, Keila Navarro-Batista, Takahiro Furuta, Masahiko Watanabe, David I. Hughes, Andrew J. Todd

**Affiliations:** a Institute of Neuroscience and Psychology, College of Medical, Veterinary and Life Sciences, University of Glasgow, Glasgow G12 8QQ, UK; b Department of Oral Anatomy and Neurobiology, Graduate School of Dentistry, Osaka University, 1-8 Yamada-Oka, Suita, Osaka 565-0871, Japan; c Department of Anatomy, Hokkaido University School of Medicine, Sapporo 060-8638, Japan

**Keywords:** AAV, adeno-associated virus, DAPI, 4′,6-diamidino-2-phenylindole, eGFP, enhanced green fluorescent protein, GRP, gastrin releasing peptide, NKB, neurokinin B, PAM, peptidylglycine alpha-amidating monooxygenase, PKCγ, protein kinase Cγ, PPD, preprodynorphin, PPTB, preprotachykinin B, TSA, tyramide signal amplification, VGAT, vesicular GABA transporter, excitatory interneuron, substance P, gastrin releasing peptide, neurokinin B, neurotensin, PKCγ

## Abstract

Around 75% of neurons in laminae I-II of the mouse dorsal horn are excitatory interneurons, and these are required for normal pain perception. We have shown that four largely non-overlapping excitatory interneuron populations can be defined by expression of the neuropeptides neurotensin, neurokinin B (NKB), gastrin-releasing peptide (GRP) and substance P. In addition, we recently identified a population of excitatory interneurons in glabrous skin territory that express dynorphin. The calcium-binding protein calretinin is present in many excitatory neurons in this region, but we know little about its relation to these neuropeptide markers. Here we show that calretinin is differentially expressed, being present in the majority of substance P-, GRP- and NKB-expressing cells, but not in the neurotensin or dynorphin cells. Calretinin-positive cells have been implicated in detection of noxious mechanical stimuli, but are not required for tactile allodynia after neuropathic pain. Our findings are therefore consistent with the suggestion that neuropathic allodynia involves the neurotensin and/or dynorphin excitatory interneuron populations. Around a quarter of inhibitory interneurons in lamina I-II contain calretinin, and recent transcriptomic studies suggest that these co-express substance P. We confirm this, by showing that inhibitory Cre-expressing cells in a Tac1^Cre^ knock-in mouse are calretinin-immunoreactive. Interestingly, there is evidence that these cells express low levels of peptidylglycine alpha-amidating monooxygenase, an enzyme required for maturation of neuropeptides. This may explain our previous finding that although the substance P precursor preprotachykinin A can be detected in some inhibitory interneurons, very few inhibitory axonal boutons are immunoreactive for substance P.

## Highlights

• Calretinin is differentially expressed among excitatory interneurons in the superficial dorsal horn of mouse spinal cord.
• Calretinin is present in many excitatory interneurons that express neurokinin B, substance P or gastrin-releasing peptide.
• Consistent with recent transcriptomic data, we find that most inhibitory substance P-expressing neurons contain calretinin.

## Introduction

The superficial dorsal horn of the spinal cord (laminae I-II) is innervated by primary afferents that respond to a variety of sensory modalities (Todd, 2010, Braz et al., 2014). Although some lamina I neurons project to the brain via the anterolateral tract, the vast majority (∼99%) of neurons in this region have axons that remain within the spinal cord, and are therefore defined as interneurons (Abraira and Ginty, 2013, Todd, 2017). The high ratio of interneurons to projection cells indicates that a considerable amount of sensory processing occurs within the superficial dorsal horn, consistent with the suggestion that neuronal circuits in this region are involved in gating pain and suppressing itch (Melzack and Wall, 1965, Braz et al., 2014). However, our understanding of the neuronal circuitry that underlies somatosensory processing remains limited, largely due to the difficulty in defining specific populations among the interneurons.

Interneurons in the superficial dorsal horn (laminae I-II) can be divided into two broad classes. In the mouse, around 75% are excitatory (glutamatergic), with the remainder being inhibitory (GABA/glycinergic) cells (Polgár et al., 2013). However, there is considerable heterogeneity within each of these classes and there have been numerous attempts to divide them into functional populations, based on various morphological, electrophysiological and neurochemical parameters (Grudt and Perl, 2002, Graham et al., 2007, Todd, 2017). Within laminae I-II, we have identified four largely non-overlapping populations of excitatory interneurons that express the neuropeptides neurotensin, neurokinin B (NKB), gastrin-releasing peptide (GRP) and substance P (Gutierrez-Mecinas et al., 2016, Gutierrez-Mecinas et al., 2017). The neurotensin and NKB cells are located in lamina IIi (as well as lamina III) and often co-express PKCγ, which is found on many excitatory interneurons in this region (Malmberg et al., 1997, Polgár et al., 1999). In contrast, the GRP and substance P cells, are located more dorsally and generally lack PKCγ. We have also identified a population of excitatory interneurons that contain the dynorphin precursor preprodynorphin (PPD), and found that these are largely restricted to regions of superficial dorsal horn that are innervated from glabrous skin (Boyle et al., 2017, Huang et al., 2018). The results of recent transcriptomic studies involving single-cell RNA sequencing are generally consistent with these findings (Haring et al., 2018, Sathyamurthy et al., 2018).

The calcium-binding protein calretinin is expressed by many superficial dorsal horn interneurons (Ren and Ruda, 1994), and two behavioral studies have implicated these cells in the processing of mechanosensory information from the skin (Duan et al., 2014, Peirs et al., 2015). It has been reported that ∼30% of neurons in laminae I-II are calretinin-immunoreactive, with the great majority being excitatory interneurons (Smith et al., 2015, Smith et al., 2016). The first aim of this study was therefore to investigate calretinin expression among the different neuropeptide-containing excitatory interneuron populations, in order to facilitate interpretation of these behavioral findings (Duan et al., 2014, Peirs et al., 2015).

Relatively little is known about the inhibitory calretinin cells, except that they correspond to a morphological class known as islet cells (Smith et al., 2015). Interestingly, transcriptomic studies have suggested that some of these cells express the mRNA for Tac1, the gene that encodes substance P (Haring et al., 2018, Sathyamurthy et al., 2018). We have previously shown that some Tac1-expressing neurons in laminae I-II are inhibitory, although surprisingly we failed to find significant numbers of inhibitory axonal boutons with substance P-immunoreactivity in the dorsal horn (Gutierrez-Mecinas et al., 2017). We therefore investigated calretinin expression among the inhibitory Tac1 cells.

We find that calretinin is differentially expressed among the peptide-expressing populations of excitatory interneurons in the superficial dorsal horn, and confirm co-expression of calretinin and Tac1 in inhibitory interneurons in this region.

## Experimental procedures

### Animals

All experiments were approved by the Ethical Review Process Applications Panel of the University of Glasgow, and were performed in accordance with the European Community directive 86/609/EC and the UK Animals (Scientific Procedures) Act 1986.

We used two genetically modified mouse lines in this study. The first of these was a BAC transgenic Tg(GRP-EGFP) from GENSAT in which enhanced green fluorescent protein (eGFP) is expressed under control of the GRP promoter (Gong et al., 2003, Gutierrez-Mecinas et al., 2014, Solorzano et al., 2015). This was used because GRP-expressing neurons cannot be detected with immunocytochemistry due to low levels of peptide expression in their cell bodies and lack of specificity of GRP antibodies (Gutierrez-Mecinas et al., 2014). We have recently shown that virtually all eGFP-positive cells in this line possess GRP mRNA, although the mRNA is also found in some cells that lack eGFP (Dickie et al., 2018). The other was a line in which Cre recombinase is inserted into the *Tac1* locus (Tac1-IRES2-Cre-D; Jackson Laboratory, Bar Harbor, ME; Stock number 021877) (Harris et al., 2014). We have shown that injection of viruses coding for Cre-dependent expression cassettes in this line labels Tac1-expressing spinal neurons (Gutierrez-Mecinas et al., 2017, Gutierrez-Mecinas et al., 2018, Dickie et al., 2018). These two lines are referred to as GRP::eGFP and Tac1^Cre^, respectively. GRP::eGFP mice were maintained as heterozygotes, while the Tac1^Cre^ mice were homozygous for this mutation.

Five adult C57BL/6 mice of either sex (18–25 g) and 3 GRP::eGFP mice of either sex (22–31 g) were deeply anesthetized with pentobarbitone (20 mg i.p.) and perfused through the left cardiac ventricle with a fixative consisting of 4% freshly depolymerized formaldehyde in phosphate buffer. Lumbar spinal cord segments were removed and post-fixed for 2 h at 4 °C in the same fixative. Tissue from these mice was cut into 60-μm-thick transverse sections with a vibrating blade microtome, and these were processed with immunocytochemistry to allow identification of interneurons belonging to various different neurochemical populations. Tissue from the GRP::eGFP mice was used to reveal GRP-expressing cells (Mishra and Hoon, 2013, Gutierrez-Mecinas et al., 2014).

To detect neurons that express Tac1, we performed intraspinal injections of an adeno-associated virus (AAV; serotype 1) that codes for a Cre-dependent form of eGFP (AAV.flex.eGFP; Penn Vector Core, Philadelphia, PA USA), as described previously (Gutierrez-Mecinas et al., 2017, Gutierrez-Mecinas et al., 2018, Dickie et al., 2018) into 3 male Tac1^Cre^ mice (19–22 g). Briefly, the mice were anesthetized with isoflurane and received two injections of AAV.flex.eGFP (each 300 nl and containing 8.6 × 10^8^ gene copies) targeted to the right dorsal horn of the L3 and L5 segments. The virus encodes an inverted sequence for eGFP between pairs of heterotypic LoxP sites with anti-parallel orientation (Atasoy et al., 2008). In infected cells that express Cre at the time of injection, there will be permanent reversal of the coding sequence, resulting in expression of eGFP. The wound was closed, and animals were allowed to recover with appropriate analgesia (buprenorphine 0.3 mg/kg and carprofen 5 mg/kg). After an 8-day survival time, the mice were re-anesthetized and perfused with fixative, as described above. Transverse sections (60 μm thick) through the L3 injection sites were cut with a vibrating blade microtome and processed for immunocytochemistry.

### Immunocytochemistry and confocal microscopy

Spinal cord sections from all animals were immersed for 30 min in 50% ethanol to enhance antibody penetration and reacted for multiple-labeling immunofluorescence staining as described previously (Gutierrez-Mecinas et al., 2014, Gutierrez-Mecinas et al., 2016). Details of the antibodies used in this study, including the sources and concentrations, are provided in Table 1. The sections were incubated for 3–5 days at 4 °C in primary antibodies diluted in PBS that contained 0.3 M NaCl, 0.3% Triton X-100 and 5% normal donkey serum, and then overnight in species-specific secondary antibodies (Jackson Immunoresearch, West Grove, PA, USA) that were raised in donkey and conjugated to Alexa 488, Alexa 647, Rhodamine Red, Pacific Blue or biotin. All secondary antibodies were diluted 1:500 (in the same diluent), apart from those conjugated to Rhodamine Red and Pacific Blue, which were diluted 1:100 and 1:200, respectively. Biotinylated secondary antibodies were detected either with Pacific Blue conjugated to avidin (1:1000; Life Technologies, Paisley, UK) or with a tyramide signal amplification (TSA) method (TSA kit tetramethylrhodamine NEL702001, PerkinElmer Life Sciences, Boston, MA, USA). The TSA reaction was used to detect antibodies directed against PPD and the NKB precursor, preprotachykinin B (PPTB), as this method can reveal the cell bodies of dorsal horn neurons that express dynorphin and NKB, respectively (Gutierrez-Mecinas et al., 2016, Boyle et al., 2017). Sections were mounted in anti-fade medium and stored at –20 °C.

**Table 1:** Antibodies used in this study

Antibody	Species	Catalog no	Dilution	Source
Calretinin	Goat	CG1	1:1000	Swant
Neurotensin	Rat		1:5000	P Ciofi
PPTB	Guinea pig		*1:5000	T Furuta
eGFP	Chicken	ab13970	1:1000	Abcam
Pax2	Rabbit	716,000	1:1000	Life Technologies
NeuN	Mouse	MAB377	1:500	Merck
NeuN	Guinea pig	266 004	1:500	Synaptic Systems
PKCγ	Rabbit	sc211	1:500	Santa Cruz Biotechnology
PKCγ	Guinea pig		1:500	M Watanabe
PPD	Guinea pig		*1:5000	T Furuta

* For tyramide signal amplification.

Sections from the L2 and L3 segments of wild-type mice were reacted with four different combinations of primary antibodies: (1), calretinin, Pax2 and NeuN (mouse antibody); (2) calretinin, neurotensin, PKCγ (guinea-pig antibody) and NeuN (mouse antibody); (3) calretinin, PPTB, PKCγ (rabbit antibody) and NeuN (mouse antibody); (4) calretinin, PPD, Pax2 and NeuN (mouse antibody). Sections processed with the first antibody combination were counterstained with 4′,6-diamidino-2-phenylindole (DAPI) to reveal cell nuclei. The sections from the GRP::eGFP mice were reacted with antibodies against calretinin, eGFP and Pax2, while those from the Tac1^Cre^ mice were reacted with antibodies against calretinin, Pax2 and NeuN (guinea pig antibody).

Sections were scanned with a Zeiss LSM 710 confocal microscope that was equipped with Argon multi-line, 405-nm diode, 561-nm solid state and 633-nm HeNe lasers. Confocal image stacks consisting of at least 25 optical sections (with a z-separation of 1 μm), were obtained through a 40× oil-immersion lens (numerical aperture 1.3) with the confocal aperture set to 1 Airy unit or less. Since excitatory dynorphin cells are largely restricted to the medial parts of the L4-5 segments, which are innervated from glabrous skin (Boyle et al., 2017, Huang et al., 2018), only this region was scanned in the sections reacted for PPD, calretinin, Pax2 and NeuN. In all other cases, the scans included the entire mediolateral extent of the dorsal horn.

### Analysis

**Fig. 1:**
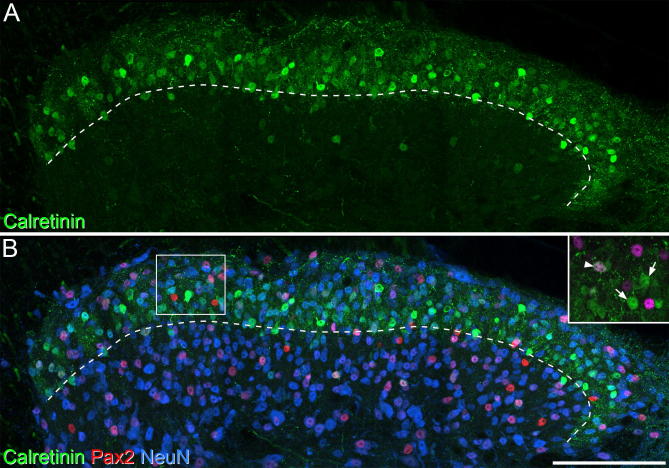
The distribution of calretinin- and Pax2-immunoreactive neurons in superficial dorsal horn. (A) Calretinin-immunoreactive neurons (green) are highly concentrated in the superficial laminae. The approximate position of the lamina II-III border is shown as a dashed line. (B) Immunoreactivity for Pax2 (shown in red), a marker for inhibitory interneurons, is distributed throughout the dorsal horn, while NeuN (shown in blue) is expressed by all dorsal horn neurons. The inset (corresponding in position to the box in B) shows staining for calretinin (green) and Pax2 (magenta). Two calretinin-immunoreactive cells that lack Pax2 are shown with arrows, and a cell that is immunoreactive for both calretinin and Pax2 is indicated with an arrowhead. The main images represent projections of 16 optical sections at 1 μm z-spacing, and the inset is taken from 4 of the optical sections. Scale bar = 100 μm. (For interpretation of the references to color in this figure legend, the reader is referred to the web version of this article.)

Confocal z-stacks were analyzed with Neurolucida for Confocal software (MBF Bioscience, Williston, VT, USA). The analysis was restricted to the superficial dorsal horn (laminae I and II), and the border between laminae II and III was identified by the much lower density of staining for calretinin in lamina III (Fig. 1). For each antibody combination, analyses were performed on between 2 and 7 sections from each of 3 animals.

Sections from wild-type mice reacted by the first antibody combination (calretinin, Pax2, NeuN) were analyzed with a stereological method to determine the proportion of neurons in laminae I and II that were calretinin-immunoreactive, and the proportion of these that were inhibitory (Pax2-positive). The NeuN and DAPI channels were first viewed, and an outline of the dorsal horn was drawn. Reference and look-up sections were set 10 μm apart, and all confocal optical sections between these were examined. The locations of all neurons in laminae I-II that had the lower surface of their nucleus between reference and look-up sections were plotted (Polgár et al., 2004). The calretinin and Pax2 channels were then viewed and the presence or absence of each type of immunostaining was recorded for each of the selected neurons.

For the other three antibody combinations performed on wild-type tissue, we initially determined the proportion of neuropeptide-expressing cells that were calretinin-immunoreactive. This was done by switching off the channel corresponding to calretinin and then identifying cells that were immunoreactive for neurotensin, PPTB or PPD throughout the depth of the z-stack. Neuropeptides and their precursor proteins can be detected in the form of granules or clumped reaction product in the perikaryal cytoplasm of neurons in which they are expressed. Neurons were defined as positive if the neuropeptide- or precursor-immunoreactivity was detected in the soma in more than one consecutive confocal optical section. In order to avoid over-sampling, we included cells if at least part of the nucleus (revealed with NeuN) was present in the first optical section in the z-series and excluded them if part of the nucleus was present in the last optical section (Cameron et al., 2015). In the case of PPD, we only analyzed excitatory PPD-immunoreactive cells (identified by lack of Pax2-immunoreactivity), because it has been reported that galanin-expressing cells, which largely correspond to the inhibitory dynorphin population (Brohl et al., 2008, Kardon et al., 2014), are not calretinin-immunoreactive (Smith et al., 2015), and as noted above this analysis was restricted to the medial part of the L4-5 segments. Having identified neuropeptide-positive neurons in laminae I-II, we then revealed the calretinin channel and determined whether each of the selected cells was calretinin-immunoreactive. During the course of this analysis, we found that PPTB, but not neurotensin or PPD, showed extensive co-localization with calretinin. In order to determine the proportion of calretinin cells that were PPTB-immunoreactive, we used a stereological method (as described above) on these sections. All neurons that had their bottom surfaces between the reference and look-up sections (which were set 10 μm apart) were identified, while only the NeuN channel was visible. The remaining channels were then viewed, and the presence or absence of calretinin, PPTB and PKCγ were recorded for each neuron in the disector sample. This part of the analysis also revealed the proportion of calretinin-immunoreactive cells that were PKCγ-immunoreactive and *vice versa*.

In sections from the GRP::eGFP mice, all of the eGFP^+^ cells within the z-series were initially identified, and the presence or absence of calretinin-immunoreactivity was then recorded for each cell. To determine the proportion of calretinin cells that were eGFP-positive, we then set reference and lookup sections 20 μm apart, viewed all of the intervening optical sections, selected all calretinin-immunoreactive cells whose bottom surface lay within this volume and recorded the presence or absence of eGFP.

In the case of Tac1-expressing neurons, we found that there was a population of inhibitory cells that co-expressed calretinin, consistent with the findings of recent transcriptomic studies (Haring et al., 2018, Sathyamurthy et al., 2018). We therefore analyzed calretinin immunoreactivity among both excitatory (Pax2-negative) and inhibitory (Pax2-positive) Tac1-expressing neurons. Initially, we performed a stereological analysis by selecting neurons (based on NeuN expression) that had their bottom surface between reference and look-up sections set 10 μm apart. We then noted the presence or absence of calretinin, eGFP and Pax2. In this way, we determined the proportion of excitatory (Pax2-negative) eGFP cells that were calretinin-immunoreactive, and the proportion of excitatory calretinin-immunoreactive cells that were labeled with eGFP. Because inhibitory neurons represent a minority among both calretinin- and Tac1-expressing cells, the numbers identified in this analysis were relatively low. We therefore analyzed inhibitory cells within the full depth of the z-stacks, rather than using a stereological method. All Pax2-immunoreactive cells within the z-stack were selected and these were then examined for the presence of calretinin and/or eGFP. In this way, we were able to determine the proportion of inhibitory calretinin cells that were eGFP-labeled, and the proportion of inhibitory eGFP cells that were calretinin-immunoreactive.

### Characterization of antibodies

The sources and dilutions of primary antibodies used in the study are listed in Table 1. The calretinin antibody was produced against recombinant human calretinin and shows no staining in the brains of calretinin-knockout mice (manufacturer's specification). Staining with the rat antiserum against neurotensin in the rat brain is identical to that seen with a well-characterized rabbit antiserum, and is blocked by pre-incubation with neurotensin (Porteous et al., 2011). The PPTB antibody was raised against amino acids 95–116 of the rat PPTB and detects PPTB (but not substance P, neurokinin A or NKB) on dot blots. Immunostaining is blocked by pre-incubation with the peptide against which the antibody was raised (Kaneko et al., 1998). The eGFP antibody was raised against recombinant full-length eGFP, and its distribution matched that of native eGFP fluorescence. The Pax2 antibody was raised against amino acids 188–385 of the mouse protein, and recognizes bands of the appropriate size on Western blots of mouse embryonic kidney (Dressler and Douglass, 1992). The mouse monoclonal antibody NeuN reacts with a protein in cell nuclei extracted from mouse brain (Mullen et al., 1992), which has subsequently been identified as the splicing factor Fox-3 (Kim et al., 2009). The guinea-pig NeuN antibody was raised against a recombinant protein consisting of amino acids 1–97 of Fox-3 and immunostains the same cells as the mouse antibody (Larsson, 2017). The two PKCγ antibodies are raised against a peptide sequence from the C terminus of mouse PKCγ. Immunostaining with the guinea pig antibody is absent from the brains of PKCγ knockout mice (Yoshida et al., 2006). The PPD antibody is directed against amino acids 229–248 at the C terminus of rat PPD, and labels PPD but not dynorphin or enkephalin. Staining is blocked by pre-incubation with the immunizing peptide (Lee et al., 1997).

## Results

### Quantification of calretinin-immunoreactive neurons in laminae I-II

The distribution of calretinin-immunoreactive neurons in mouse dorsal horn was similar to that described previously (Peirs et al., 2015, Smith et al., 2015, Smith et al., 2016). Immunoreactive cells were most densely packed in lamina II, but were also present in moderate numbers in lamina I (Fig. 1). They were far less numerous in deeper dorsal horn laminae, although scattered cells were present, particularly in lamina V.

Between 377 and 545 (mean 463) lamina I-II neurons, identified by expression of NeuN, were identified in the 3 mice used for stereological analysis of calretinin and Pax2. Smith et al. (2016) had reported that ∼30% of superficial dorsal horn neurons were calretinin-immunoreactive, and that 15% of these were Pax2-positive (i.e. inhibitory). We found slightly higher proportions in both cases, since our analysis revealed that 38.6% (range 35–43.6%) of all neurons in laminae I and II were labeled with the calretinin antibody, while 16.7% (14.7–18.8%) of these cells were Pax2-immunoreactive. Calretinin-immunoreactivity was detected in 42% (37.2–48.9%) of Pax2-negative (excitatory) neurons and in 27.4% (26.8–28.1%) of Pax2-positive (inhibitory) neurons.

### The extent of co-expression of calretinin with neurotensin, PPTB, PKCγ and PPD

**Fig. 2:**
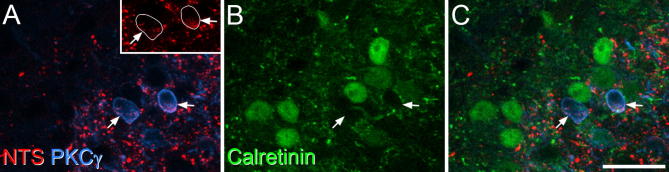
Lack of calretinin in neurons that express neurotensin. (A) A field from the inner part of lamina II scanned to reveal neurotensin (NTS, red) and PKCγ (blue). Arrows point to two PKCγ-immunoreactive neurons, both of which contain granules of neurotensin-immunoreactivity in their perikaryal cytoplasm. The inset shows the same field, with only neurotensin-immunoreactivity revealed. The outlines of the two cells have been superimposed. (B) The same field scanned to reveal calretinin (green). (C) The merged image shows that the neurotensin-positive cells lack calretinin-immunoreactivity, although they are surrounded by several calretinin-immunoreactive cells. The images are projections of 4 optical sections at 1-μm z-spacing. Scale bar = 20 μm. (For interpretation of the references to color in this figure legend, the reader is referred to the web version of this article.)

As described in previous studies, both neurotensin- and PKCγ-immunoreactive cells were concentrated in the inner part of lamina II, and were also present in lamina III (Gibson et al., 1981, Hunt et al., 1981, Mori et al., 1990, Yoshida et al., 1990, Malmberg et al., 1997). The laminar distribution for both types of immunostaining was identical to that described previously for the mouse lumbar spinal cord (see Fig. 2 of Gutierrez-Mecinas et al., 2016). The mean number of neurotensin-positive cells analyzed in lamina II was 62 per mouse, and consistent with our previous report (Gutierrez-Mecinas et al., 2016) we found that all but four of these cells (98%) were also PKCγ-immunoreactive. Even though they were surrounded by numerous calretinin-immunoreactive cells, none of the neurotensin-positive cells were labeled with the calretinin antibody (Table 2, Fig. 2).

**Table 2:** Proportions of different excitatory interneuron populations that were calretinin-immunoreactive

	Number of neurons examined in each population	% of these neurons that are CR-immunoreactive	% of CR-immunoreactive neurons that belong to each population
NTS+	61.7 (51–69)	0% (0%)	
PPTB+	50.3 (48–53)	84.0% (79.2–86.8%)	20.8% (19.7–22.5%)
PKCγ+	89.3 (71–104)	49.5% (46.2–55.9%)	21.7% (17.6–25.1%)
PKCγ+/PPTB−	64 (48–75)	34.4% (29.2–42.0%)	10.8% (7.5–12.6%)
PPD+/Pax2−	30.7 (23–39)	5% (0–10%)	
GRP-eGFP	217 (174–256)	55% (48.9–61.5%)	18.9% (17.1–21.1%)
Tac1+/Pax2−	104 (90–130)	73.2% (71–75.6%)	34.5% (29.3–41.1%)

The second column shows the numbers of cells examined per mouse among each of the classes listed in column 1, and the third column shows the proportion of these that were calretinin-immunoreactive. In some cases, the proportion of laminae I-II calretinin-immunoreactive cells that were also immunoreactive for the other marker was determined, and this is shown in the fourth column. Ranges are in brackets.

**Fig. 3:**
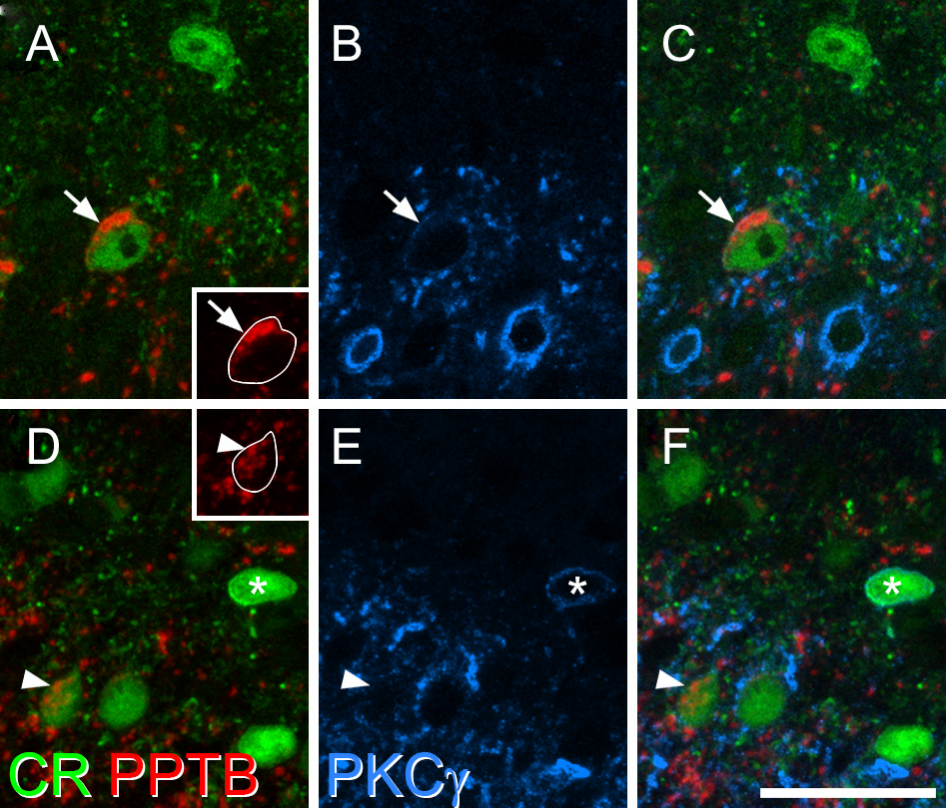
Expression of calretinin in neurons that are immunoreactive for preprotachykinin B (PPTB) and/or PKCγ. (A) A field scanned for calretinin (green) and PPTB (red) shows co-localization of both proteins in a neuron in the inner part of lamina II (arrow). (B) the scan for PKCγ (blue) shows that this cell is weakly immunoreactive. (C) A merged image. (D-F) Corresponding scans from a nearby region include a neuron that is immunoreactive for both PPTB and calretinin, but lacks PKCγ (arrowhead), as well as a PKCγ cell that is calretinin-immunoreactive but lacks PPTB (asterisk). Insets in **A** and **D** show the staining for PPTB with the outlines of the cells superimposed. The images in **A**-**C** are from a single optical section, while those in **D**-**F** are from three optical sections at 1 μm z-spacing. Scale bar = 20 μm. (For interpretation of the references to color in this figure legend, the reader is referred to the web version of this article.)

PPTB-immunoreactive cells showed a similar laminar distribution to that of the neurotensin cells, as reported previously (Fig. 3 of Gutierrez-Mecinas et al., 2016). They were present in the inner part of lamina II, extending into lamina III. Consistent with our previous results, we found that 50.4% (45.3–58%) of these cells were PKCγ-immunoreactive, although these generally showed weak staining with the PKCγ antibody. PPTB cells accounted for 28.7% (25.8–32.4%) of the PKCγ cells in laminae I-II. Comparison with calretinin immunostaining showed that 84% of the PPTB cells were calretinin-immunoreactive, and that these accounted for 21% of the calretinin cells in the superficial dorsal horn (Table 2, Fig. 3). We also analyzed calretinin expression among all PKCγ-immunoreactive cells in laminae I-II in these sections, and found that 50% of PKCγ-positive cells contained calretinin, accounting for 22% of the calretinin-positive cells in this region (Table 2, Fig. 3).

Our previously published immunocytochemical data (Gutierrez-Mecinas et al., 2016) indicate that PKCγ-expressing cells in the superficial dorsal horn can be divided into three groups of approximately equal size: those with neurotensin, those with PPTB and those that do not express either peptide. Since we had found that the neurotensin cells were never calretinin-immunoreactive, any expression of calretinin among PKCγ cells that lacked PPTB must be restricted to this latter population (those that do not express neurotensin or PPTB). We found that 34% of PKCγ cells that lacked PPTB were calretinin-immunoreactive, and that these accounted for 11% of calretinin cells. An example is shown in Fig. 3d-f. Most of these cells showed very weak staining for PKCγ.

**Fig. 4:**
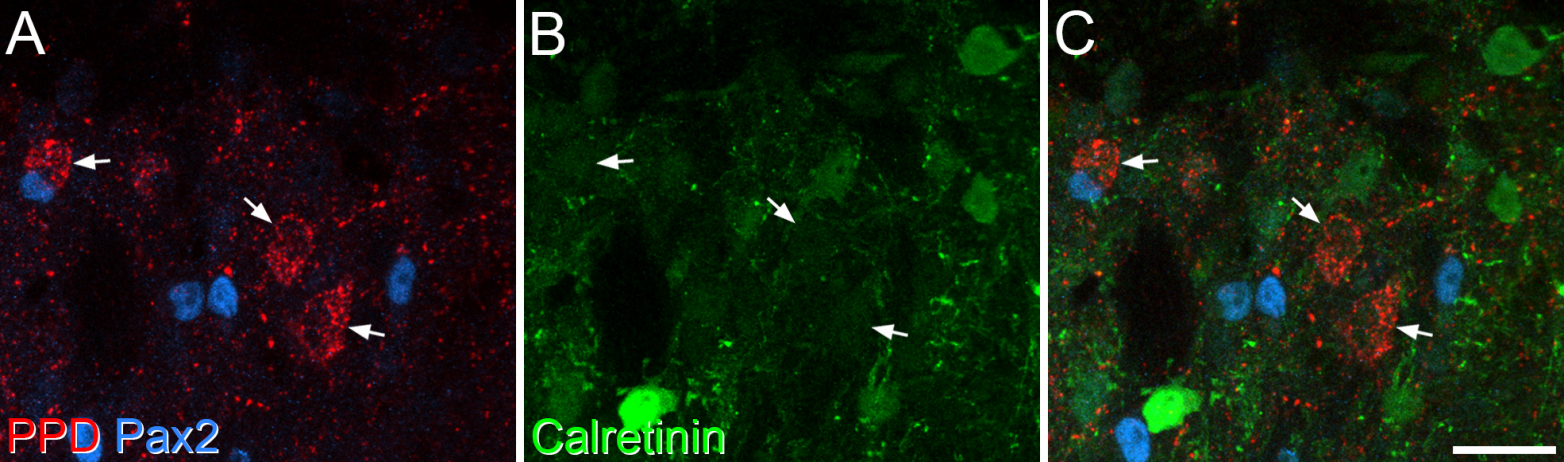
Lack of calretinin in excitatory neurons that express preprodynorphin (PPD). (A) A field from the medial part of the superficial dorsal horn in the L5 segment scanned to reveal PPD (red) and Pax2 (blue). Three PPD-positive/Pax2-negative cells are shown (arrows). (B) The same field scanned to reveal calretinin (green). (C) The merged image shows that the 3 Pax2-negative (excitatory) PPD cells are not calretinin-immunoreactive. Images are projections of 4 optical sections at 1-μm z-spacing. Scale bar = 20 μm. (For interpretation of the references to color in this figure legend, the reader is referred to the web version of this article.)

PPD immunoreactivity was highly concentrated in laminae I-II, as illustrated in Fig. 4 of Boyle et al. (2017). As described previously (Boyle et al., 2017, Huang et al., 2018), we found that PPD-immunoreactive cells that lacked Pax2 (i.e. excitatory dynorphin-expressing neurons) were concentrated in the medial part of laminae I-II in the L4-L5 segments. The great majority of these cells were not immunoreactive for calretinin (Fig. 4), however, very weak calretinin immunostaining was detected in 5% of these cells (Table 2).

### Co-expression of calretinin with eGFP in the GRP-eGFP mouse

**Fig. 5:**
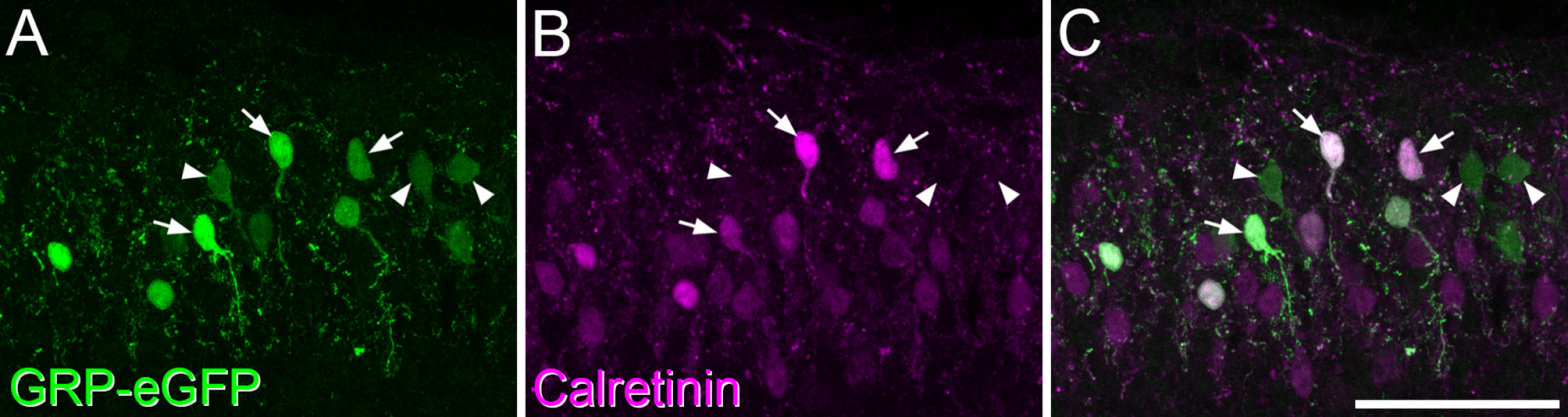
Expression of calretinin by some eGFP-positive neurons in the GRP::eGFP mouse. (A) A field that includes part of lamina II scanned to reveal eGFP (green). Several labeled neurons are visible, and some of these are indicated with arrows or arrowheads. (B) The same field scanned for calretinin (magenta). (C) The merged image shows that some of the eGFP-positive cells are calretinin-immunoreactive (arrows), while others are calretinin-negative (arrowheads). Images are projections of 7 optical sections at 1-μm z-spacing. Scale bar = 50 μm. (For interpretation of the references to color in this figure legend, the reader is referred to the web version of this article.)

In tissue from the GRP::eGFP mouse, eGFP-positive cells were concentrated in lamina II (as shown in Fig. 6 of Gutierrez-Mecinas et al., 2014). We have previously reported that eGFP cells are present at relatively low density in regions of the superficial dorsal horn that receive their primary afferent input from glabrous skin (the medial parts of L4-5) (Dickie et al., 2018). We therefore analyzed sections from the L2 and L3 segments, as these are innervated from hairy skin. We found that 55% of the eGFP-positive cells were calretinin-immunoreactive, and that these accounted for 19% of calretinin cells (Table 2, Fig. 5).

### Calretinin expression in excitatory and inhibitory Tac1 cells

**Fig. 6:**
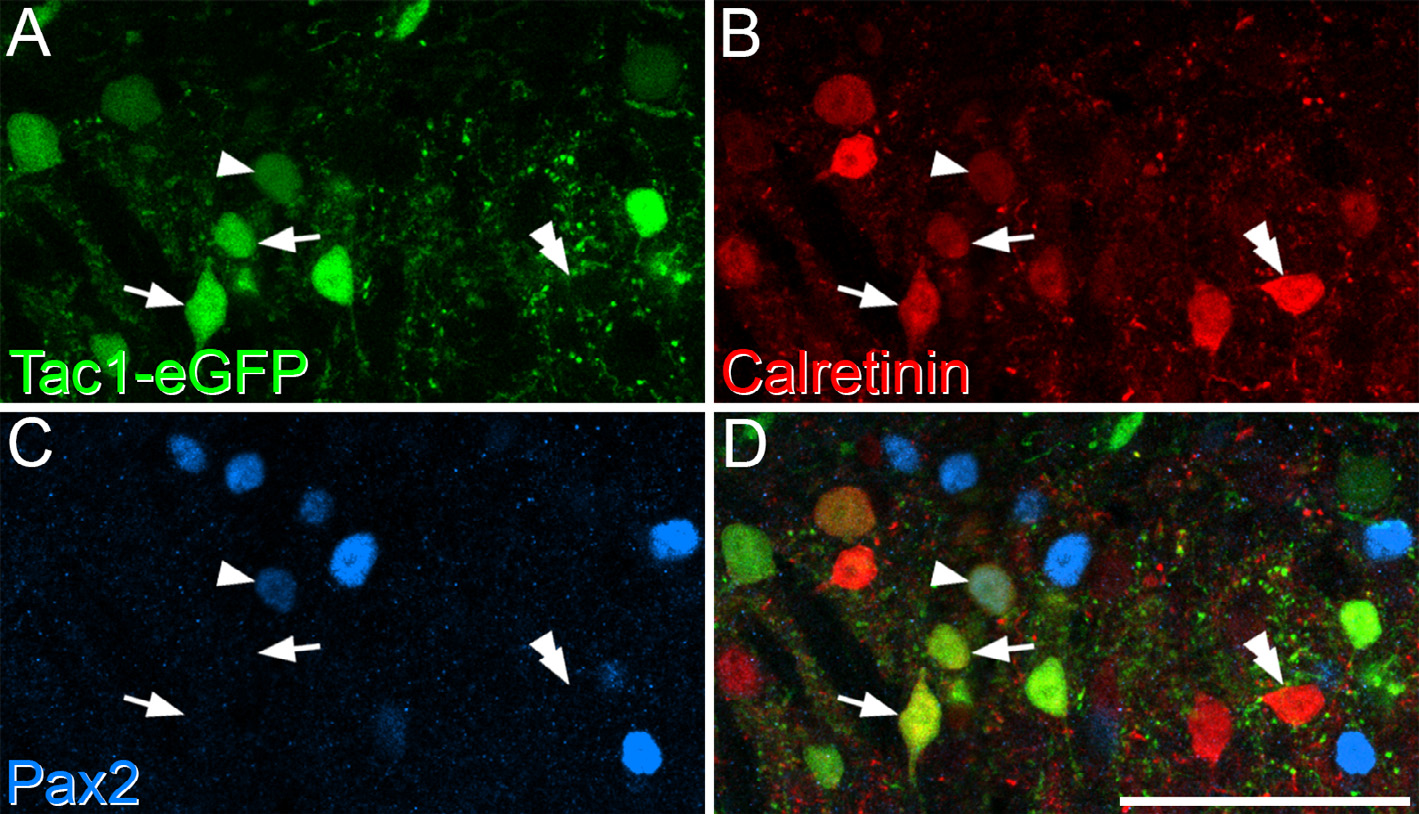
Calretinin and Pax2 expression in tissue from a Tac1^Cre^ mouse that had received an intraspinal injection of AAV.flex.eGFP. (A) Part of lamina II within the injected region of the dorsal horn, showing eGFP (green) in Tac1-positive cells. (B, C) The same field scanned to reveal calretinin (red) and Pax2 (blue). (D) The merged image shows that some Pax2-negative/eGFP-positive (excitatory Tac1) cells are calretinin-immunoreactive, and two of these are marked with arrows. A single Pax2-positive/eGFP-positive (inhibitory Tac1) cell is present, and this is also calretinin-immunoreactive (arrowhead). The double arrowhead shows an example of a calretinin-immunoreactive cell that is negative for eGFP and Pax2. Images are projections of 4 optical sections at 1-μm z-spacing. Scale bar = 50 μm. (For interpretation of the references to color in this figure legend, the reader is referred to the web version of this article.)

The expression of eGFP in the injected segments of the Tac1^Cre^ mice was very similar to that described in our previous studies following intraspinal injection of AAVs coding for Cre-dependent fluorescent proteins in these animals (e.g. Fig. 7 of Gutierrez-Mecinas et al., 2017). The labeled cells were particularly numerous in lamina II, but were also present in lamina I and scattered through the deeper dorsal horn. Consistent with our previous report, we found that 12.3% (range 9.7–16.7%) of the eGFP-labeled cells were Pax2-positive, indicating that the majority of the cells (∼88%) were excitatory neurons. Quantitative analysis revealed that 73% of eGFP-positive/Pax2-negative (excitatory Tac1) cells were calretinin-immunoreactive, and that these accounted for 35% of the calretinin cells in laminae I-II (Table 2, Fig. 6).

We also analyzed calretinin-immunoreactivity among the eGFP-positive/Pax2-positive (inhibitory Tac1) cells. We identified a mean of 59 of these cells per mouse (range 46–75) and found that virtually all of them (mean 95.3%, range 92.9–97.3%) were calretinin-immunoreactive (Fig. 6). eGFP-positive cells accounted for 36.9% (range 31.2–41%) of the inhibitory (Pax2-positive) calretinin-immunoreactive cells within laminae I-II.

## Discussion

The main findings of this study are: (1) that calretinin-immunoreactivity can be detected in ∼40% of excitatory neurons in laminae I-II and around 25% of the inhibitory cells; (2) that it is differentially distributed among the neuropeptide-expressing subsets of excitatory interneurons, being present in most of those that express substance P or PPTB, many of the GRP-eGFP cells, but very few of the dynorphin or neurotensin cells; and (3) that virtually all of the Tac1-positive inhibitory cells are calretinin-immunoreactive, with these cells accounting for over a third of the inhibitory calretinin population.

### Calretinin expression among excitatory interneuron populations

Our identification of distinct excitatory interneuron populations that express neurotensin, NKB, GRP or substance P was based on identification of neuropeptides that showed a restricted laminar distribution and were known to be expressed by glutamatergic cells (Xu et al., 2008, Gutierrez-Mecinas et al., 2016, Gutierrez-Mecinas et al., 2017). We subsequently described a population of excitatory dynorphin cells in regions innervated from glabrous skin (Boyle et al., 2017, Huang et al., 2018), although it is not yet known whether these cells overlap with the other four neuropeptide populations.

Two recent studies (Haring et al., 2018, Sathyamurthy et al., 2018) have used a transcriptomic approach to define neuronal populations within the dorsal horn based on expression of a wide range of mRNAs. Both of these studies identify populations that correspond to neurotensin-, NKB- and substance P-expressing excitatory neurons. In the study by Sathyamurthy et al, each of these corresponds to a specific cluster, named DE-6, DE-5 and DE-11, respectively. According to Haring et al, the neurotensin population corresponds to a specific cluster (Glut4), while NKB- and substance P-expressing cells are each included in more than one cluster (Glut5-7 and Glut10-11, respectively). However, it should be noted that while cells in the Glut4 (neurotensin) and Glut5-7 (NKB) clusters are largely restricted to laminae II-III, the substance P-expressing clusters (Glut10-11) both include cells with a far wider distribution, extending from laminae I-V. It is therefore likely that the population of substance P-expressing excitatory interneurons that we have identified in lamina II (Gutierrez-Mecinas et al., 2017, Gutierrez-Mecinas et al., 2018, Dickie et al., 2018) corresponds to only a part of one or both of these clusters. Although Sathyamurthy et al. also recognize GRP (DE-1) and excitatory dynorphin (DE-15) populations, these do not appear in the study by Haring et al.

Our finding that calretinin is present in high proportions of the NKB- and substance P-classes, but not among the neurotensin cells is in good agreement with the data reported by Haring et al, since their neurotensin population (Glut4) showed very low expression of the Calb2 gene (which codes for calretinin), whereas this was more highly expressed in their Glut5-7 (NKB) and Glut10-11 (Tac1) clusters. The relationship between our findings and those reported by Sathyamurthy et al. is somewhat less clear. Consistent with our results, their DE-5 (NKB) and DE-11 (Tac1) populations both show relatively high levels of Calb2 expression, while their DE-15 (dynorphin) population shows much lower expression. However, they report very low Calb2 expression for the DE-1 (GRP) population, and moderate expression for the DE-6 (neurotensin) cells, which does not fit with our finding that many GRP-eGFP cells, but no neurotensin-immunoreactive cells were calretinin-immunoreactive. It should be noted that discrepancies between the interpretation of transcriptomic and immunocytochemical findings could result from the way in which cells are clustered, and/or from differences between levels of mRNAs and the corresponding proteins in individual neurons.

Several studies have implicated PKCγ-expressing cells in the tactile allodynia that results from peripheral nerve injury (Malmberg et al., 1997, Lu et al., 2013, Peirs et al., 2015, Peirs and Seal, 2016). We previously reported that different subsets of PKCγ-immunoreactive neurons could be recognized, based on expression of neurotensin and PPTB, and this distinction is also supported by the findings of Haring et al. (2018), who detected PKCγ mRNA in Glut4 (neurotensin) and Glut5-7 (NKB), as well as in Glut2-3 (two populations that are defined by expression of cholecystokinin). Although Sathyamurthy et al. (2018) identify a specific population of PKCγ-expressing excitatory neurons (DE-4), they also detect transcripts for PKCγ in other interneuron populations, for example those defined by expression of neurotensin, PPTB and cholecystokinin (DE-5, 6 and 7, respectively). Here we provide further evidence for heterogeneity among PKCγ-expressing cells by showing that neurotensin-immunoreactive subset lack calretinin, whereas other types of PKCγ cell (including those that express PPTB) are often calretinin-positive.

Smith et al., 2015, Smith et al., 2016 characterized calretinin-expressing cells by performing whole-cell patch-clamp recording in the superficial dorsal horn of a bacterial artificial chromosome (BAC) transgenic mouse line in which eGFP is expressed under control of the calretinin promoter (Caputi et al., 2009). They identified two classes of cell, which they named typical and atypical, and showed that these corresponded to excitatory and inhibitory calretinin neurons, respectively. They reported that all typical (excitatory) calretinin cells showed an outward current following application of noradrenaline and most responded similarly to 5-hydroxytryptamine (5-HT). However, they did not respond to μ opioid agonists (Smith et al., 2016). We have recently investigated the properties of two excitatory interneuron populations, those that express GRP or substance P, and found that these had very different responses to neuromodulators (Dickie et al., 2018). All of the substance P cells tested showed outward currents in response to 5-HT and most to noradrenaline, whereas these cells were not affected by the μ opioid ligand DAMGO. In contrast, virtually all GRP cells responded to DAMGO, but few to noradrenaline and none to 5-HT. Since many substance P and GRP cells express calretinin, these are likely to have been included among the eGFP+ cells in the calretinin-eGFP mice used by Smith et al. The pharmacological profile reported in their studies would fit well with the properties of the substance P cells that we recorded. However, our data suggest that GRP-expressing cells were likely to have been under-represented in the sample they tested with neuromodulators.

### Involvement of calretinin interneurons in pain processing

Two studies have investigated the role of calretinin-expressing dorsal horn neurons in pain mechanisms. Duan et al. (2014) ablated these cells in a mouse line in which Cre recombinase was knocked into the calretinin gene and reported that this resulted in an increased threshold in the von Frey test. Peirs et al. (2015) used a chemogenetic approach to activate Cre-expressing cells in the same mouse line. Their strategy targeted a subset of calretinin-immunoreactive neurons that were located in the middle part of lamina II, and excluded those that were PKCγ-immunoreactive. Activating these cells caused a reduction of von Frey thresholds, as well as guarding of the affected paw. Together, these results indicate that calretinin cells are involved in the response to mechanical stimuli. However, Duan et al. found that ablating the calretinin cells had no effect on mechanical allodynia after nerve injury.

Peirs et al. (2015) also provided evidence that PKCγ neurons were involved in the tactile allodynia associated with peripheral nerve injury, but not in that seen in inflammatory pain states. They therefore proposed that different microcircuits were responsible for these two forms of allodynia (Peirs et al., 2015, Peirs and Seal, 2016). The circuit underlying neuropathic allodynia is thought to involve low-threshold mechanoreceptive input to PKCγ cells. These indirectly activate vertical cells in lamina IIo, which are presynaptic to nociceptive lamina I projection neurons (Lu et al., 2013). The great majority of neurotensin-positive cells in lamina IIi-III express PKCγ, and since these cells lack calretinin, they would have been spared in the ablation of calretinin cells performed by Duan et al. (2014). It is therefore possible that neurotensin-expressing cells contribute to tactile allodynia after nerve injury. Interestingly, we find that calretinin is also largely excluded from the excitatory dynorphin neurons. We recently reported that chemogenetic activation in a Pdyn^Cre^ mouse line resulted in tactile allodynia, and this was thought to involve dynorphin-expressing excitatory interneurons, some of which are vertical cells (Huang et al., 2018). It is not yet known whether these dynorphin cells are involved in allodynia resulting from neuropathic pain, but again, their lack of calretinin expression means that they could have contributed to the mechanical allodynia that Duan et al. observed after nerve injury in mice in which calretinin cells had been ablated. Future studies will need to investigate whether the excitatory dynorphin cells are necessary for the development of nerve injury-evoked allodynia.

### Tac1 expression by inhibitory calretinin cells

Smith et al. (2015) were the first to identify Pax2-expressing calretinin neurons in the superficial dorsal horn, and these were shown to be islet cells, a well-established class of inhibitory interneuron in lamina II (Todd and McKenzie, 1989, Lu and Perl, 2003, Maxwell et al., 2007, Yasaka et al., 2010). Both Sathyamurthy et al., 2018, Haring et al., 2018 identified Calb2-positive populations among the inhibitory neurons (DI-1, and Gaba8-9, respectively). The DI-1cluster showed relatively strong expression of Tac1 (Sathyamurthy et al., 2018), while a high level of expression was also seen in the Gaba9 (but not Gaba8) population (Haring et al., 2018). This therefore fits well with our finding that virtually all of the inhibitory Tac1 cells were calretinin-immunoreactive (constituting 37% of inhibitory calretinin cells in laminae I-II). These would presumably correspond to the DI-1 and Gaba9 classes.

At the time, our finding of inhibitory Tac1 neurons was surprising, because we were unable to detect substance P in axonal boutons of inhibitory interneurons, identified by the presence of the vesicular GABA transporter (VGAT) (Gutierrez-Mecinas et al., 2017). Furthermore, we were able to detect the precursor protein (preprotachykinin A, PPTA) in cell bodies of inhibitory neurons in superficial laminae, indicating that these cells were not only transcribing the Tac1 gene, but also translating the Tac1 mRNA (Gutierrez-Mecinas et al., 2017). Interestingly, Sathyamurthy et al. (2018) reported that their DI-1 population showed a low level of expression of the enzyme peptidylglycine alpha-amidating monooxygenase (PAM), which is required for the maturation of many biologically active peptides. Specifically, PAM causes α-amidation of several neuropeptides, including substance P (Eipper et al., 1993). If the inhibitory substance P cells lack this enzyme, they may not be able to generate the mature peptide from PPTA, and this could explain our failure to detect substance P in VGAT boutons.

## Conclusion

These results show that calretinin is differentially expressed among neuropeptide-containing excitatory interneuron populations in the superficial dorsal horn. The lack of calretinin in neurotensin and dynorphin excitatory interneurons is consistent with the suggestion that these cells are involved in tactile allodynia after nerve injury. We also confirm transcriptomic findings that calretinin is expressed by the inhibitory Tac1 cells.
